# Hiding in Plain Sight: A Retrospective Review of Unrecognized Tumors During Dermatologic Surgery

**DOI:** 10.7759/cureus.23487

**Published:** 2022-03-25

**Authors:** Alexander Reid, Emily Weig, Kirsten Dickinson, Faraaz Zafar, Roshan Abid, Marta VanBeek, Nkanyezi Ferguson

**Affiliations:** 1 Department of Dermatology, University of Iowa Hospitals and Clinics, Iowa City, USA; 2 Department of Dermatology, Cleveland Clinic, Cleveland, USA

**Keywords:** heuristic decision, decision making process, cognitive bias, quality improvement and patient safety, dermatology and dermatologic surgery

## Abstract

Background: Mohs micrographic surgery requires focused attention that may lead to tunnel vision bias, contributing to not recognizing skin cancer at nearby sites.

Objective: It is to determine if a subsequently diagnosed skin cancer was visible at the time of Mohs surgery.

Methods: A retrospective chart review was performed at a single academic center from 2008 to 2020. Patients who underwent at least two distinct MMS procedures, separated in time to capture subsequent tumors, were included.

Results: Four hundred and four individual patients were identified with at least two distinct Mohs procedures, which generated 1,110 Mohs sequences. Fifty-one (4.6%) clinically apparent tumors went unrecognized and 127 (11.4%) tumors were identified and biopsied during the visit. High-risk tumor histology was identified in 10 (20%) unrecognized tumors and 31 (24%) recognized tumors (p-value 0.491).

Conclusion: Our study suggests that Mohs surgeons may be overlooking adjacent skin cancers when focusing only on the tumor being surgically treated. Tunnel vision bias may account for part of this phenomenon.

## Introduction

Mohs micrographic surgery (MMS) is a specialty that requires meticulous attention to detail when taking layers, examining histologic sections, and executing reconstructive repairs. One potential consequence of this focused attention may be a phenomenon called tunnel vision bias in which the surgeon fails to notice a visible skin cancer in the same anatomic region being operated upon. Ultimately, this delay in diagnosis could result in increased morbidity, especially with high-risk tumors. This study seeks to determine if a subsequently diagnosed histologically confirmed skin cancer was visible at the time of initial Mohs surgery. We aim to determine the incidence of unrecognized tumors in dermatologic surgery at a single academic center.

The psychologist and economist Daniel Kahneman, known for his pioneering research into cognitive psychology and decision-making, divide the mental functioning that undergirds decision-making and judgment into two separate systems [1]. System 1 relies on intuition to automatically solve problems while System 2 relies on reflective thinking to reason through complex problems [1]. System 1 uses heuristics (mental shortcuts) to find quick and easy answers [1]. Heuristics allow for the rapid processing of information to effectively and efficiently interact with the environment, especially when little relevant information is available. When improperly applied, heuristics give rise to cognitive bias which can result in error [1-3]. Tunnel vision, a form of cognitive bias, is the mental process by which humans physically see something but fail to perceive that it is present on a conscious level [4]. Therefore, tunnel vision bias causes humans to misinterpret their environment. In the medical field, this misinterpretation can lead to medical errors or missed diagnoses.

Medical errors are estimated to occur up to 400,000 times each year, which may place them as the third leading cause of mortality in the United States [3,5-7]. Their estimated cost of $20 billion annually burdens an already fiscally challenged medical system [7]. Medical errors are divided into diagnostic, treatment, preventative, and other errors [3]. Tunnel vision bias contributes to diagnostic errors (misdiagnosis, missing a diagnosis, etc.). Importantly, the leading cause of medical malpractice claims is diagnostic errors [8,9].

The critical role of cognitive biases is well established in the psychology, legal and medical literature [1,2,10]. Tunnel vision bias was studied in the field of radiology by researchers placing a cartoon gorilla into the images of a chest computed tomography (CT) scan. Radiologists were instructed to identify pulmonary nodules when reading these scans. After interpreting the CT scan, a full 83% of radiologists were completely oblivious to the cartoon gorilla inserted within the images [11]. Although this study may humorously point out the power of tunnel vision bias, the real-world implications are substantial. In fact, tunnel vision bias is the fourth most common cause of diagnostic errors in radiology [12]. Interestingly, tunnel vision bias may overlap with anchoring bias (locking onto initial information while failing to adjust for new information) if clinicians place too much weight on the initial task such as finding pulmonary nodules or taking a Mohs layer [3].

There is a need to further explore the role that cognitive heuristics (mental shortcuts) and bias play in dermatologic decision-making [2]. In dermatology, heuristics allow for a visual diagnosis to be made in as little as 200 milliseconds [13]. One of the most relied upon and least biased heuristics in the field of dermatology is the primary lesion heuristic [4]. This heuristic relies on information gleaned only from the nature and character of a primary lesion as opposed to other pieces of evidence. The apple jelly appearance of a lesion immediately summons to mind a diagnosis of sarcoidosis [4]. Cognitive bias affects the visual system, which directly impacts dermatology. The perception of a colored lesion is influenced by which other colors are surrounding it. For example, two identical shades of gray appear different when one is framed with a dark background and the other with a light background [14]. As a corollary, dermatologists may misperceive erythema in the skin of color patients because the background skin tone is darker [4,14]. Despite the recognition of tunnel vision bias in other medical specialties, no research has been done to quantify its effects on dermatologic surgeons [15]. Our study seeks to narrow this knowledge gap.

## Materials and methods

**Figure 1:**
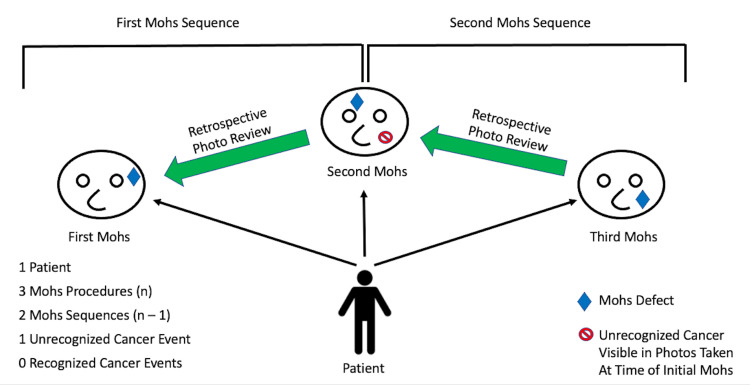
Outline of the method used to determine the number of Mohs sequences generated by an example patient’s chart. Photographs of the defect at the subsequent Mohs procedure were compared to photographs taken at the time of the prior Mohs procedure to determine if the defect corresponded to a visible but unrecognized skin lesion.

A retrospective chart review was performed at a single, tertiary academic referral center. Electronic medical records from January 1, 2008 to September 30, 2020 were searched. During our study period, a total of 13,566 Mohs operations were performed. Four hundred and four patients who underwent at least two MMS operations, separated in time, were included which generated 1,110 Mohs sequences (Figure 1). A single Mohs sequence was defined as an MMS patient encounter on any given date that also had another distinct Mohs procedure that occurred temporally later. Under this definition, a single unique patient could have generated multiple Mohs sequences (Figure 1). Therefore, if a single patient had (n) number of Mohs procedures, the total number of Mohs sequences generated by that individual patient was (n) minus 1 (Mohs Sequence = n - 1) (1 represents the original Mohs surgery). Routine perioperative photographs taken at the time of MMS were compared to photographs of subsequently biopsied skin cancers and reviewed for evidence of this tumor at the time of initial Mohs surgery.

Consequently, only subsequent tumors that were in the same or adjacent anatomic region as the original surgical site were evaluated. Routine perioperative photographs of the surgical site are typically performed from three different angles (head-on, left side, right side) with a fourth inferior angle occasionally employed. Two independent reviewers viewed the photos and a third reviewer arbitrated non-concordant cases. Tumors were categorized as either unrecognized or recognized cancer events at the time of surgery. Any cancerous lesion biopsied on the day of MMS was considered a recognized cancer event. In the authors’ clinical practice when lesions of concern are noted at the time of Mohs surgery, they are biopsied as clinically indicated as opposed to being discussed with the patient then sent back to the referring provider for evaluation.

Patient demographics, tumor type, months until definitive MMS and immune status were collected. Immunosuppression was defined as patients with chronic lymphocytic leukemia (CLL) or those with an organ transplant on immunosuppressive medications. High-risk tumor histopathology was defined as basal cell carcinoma (BCC) with morpheaform, basosquamous, infiltrative or micronodular features; squamous cell carcinoma (SCC) with poor or moderate differentiation; and melanoma (any subtype). Chi-squared tests were used to evaluate categorical variables including immune status, sex, and high/low-risk tumor status. Mann-Whitney U tests were used to evaluate non-parametric continuous variables such as age and Mohs intervals. All confidence levels (alpha) were 0.05 and all statistical tests were two-sided. All statistical analyses were performed using GraphPad Prism (9.1.0). This study was reviewed and approved by the University of Iowa Hospitals and Clinics Institutional Review Board (IRB).

## Results

The median age for patients in the unrecognized cancer event group was 76 years with an age range of 32 to 90 years (interquartile range 23 years) (Table 1).

**Table 1:** Characteristics between unrecognized versus recognized cancer event groups. *P-value from Mann-Whitney U test for non-parametric data †P-value from chi-square ‡ Immunosuppression includes chronic lymphocytic leukemia (CLL) and organ transplant on immunosuppressive medications § High Risk: Basal cell carcinoma: morpheaform, basosquamous, infiltrative and micronodular; squamous cell carcinoma: poorly differentiated and moderately differentiated; melanoma (any subtype)

Characteristic	Unrecognized Cancer Event		Recognized Cancer Event		P-value
Median Age in Years	76		71		0.388 *
Age Range in Years	32-90		42-93		
Male Sex	Number	Percent	Number	Percent	0.067 †
	33	65	99	78	
Immunosuppression ‡	7	14	49	39	0.001 †
High Risk §	10	20	31	24	0.491 †
Low Risk	41	80	96	76	
Median Mohs Interval in Months	High Risk §		Low Risk		0.747 *
	4		6		
Mohs Interval Interquartile Range in Months	7		7		
Range of Mohs Intervals in Months	2-18		1-28		

The median age in the recognized cancer event group was 71 years with a range of 42 to 93 years (interquartile range 18 years, p-value 0.388). There were 33 (65%) males in the unrecognized cancer event group, and 99 (78%) males in the recognized cancer event group (p-value 0.067). There were seven (14%) immunosuppressed patients in the unrecognized cancer event group, and 49 (39%) immunosuppressed patients in the recognized cancer event group (p-value 0.001).

A review of 404 patient charts generated 1,110 Mohs sequences. Of the 1,110 Mohs sequences, 51 (4.6%) tumors went unrecognized, and 127 (11.4%) tumors were successfully recognized and biopsied on the same day as the Mohs procedure. Therefore, there was a 16% (178/1,110) probability of a second, visible skin cancer being present at the time of the first Mohs surgery. There were over twice as many tumors recognized on the day of Mohs surgery compared to the number of tumors unrecognized. High-risk tumor histology was identified in 10 (20%) of the unrecognized tumors and 31 (24%) of the successfully recognized tumors (p-value 0.491) (Table 1). In the unrecognized cancer event group, 16 (33%) tumors were SCC while 34 (67%) tumors were BCC (excluding the one melanoma (unrecognized cancer event group) identified in our study). In the recognized cancer event group, 74 (58%) tumors were SCC, 46 (36%) tumors were BCC and seven (6%) Mohs sequences had both SCC and BCC identified. Excluding the seven Mohs sequences with both BCC and SCC identified concurrently, there were significantly more SCCs in the recognized cancer event group compared to the unrecognized cancer event group (74 [62%] vs. 16 [32%], p<0.001). The time interval from the initial Mohs encounter until definitive treatment of an unrecognized cancer was a median of four months (interquartile range seven months, range 2-18 months) for a high-risk tumor and a median of six months (interquartile range seven months, range 1-28 months) for a low risk tumor (p-value 0.747). 

## Discussion

**Figure 2:**
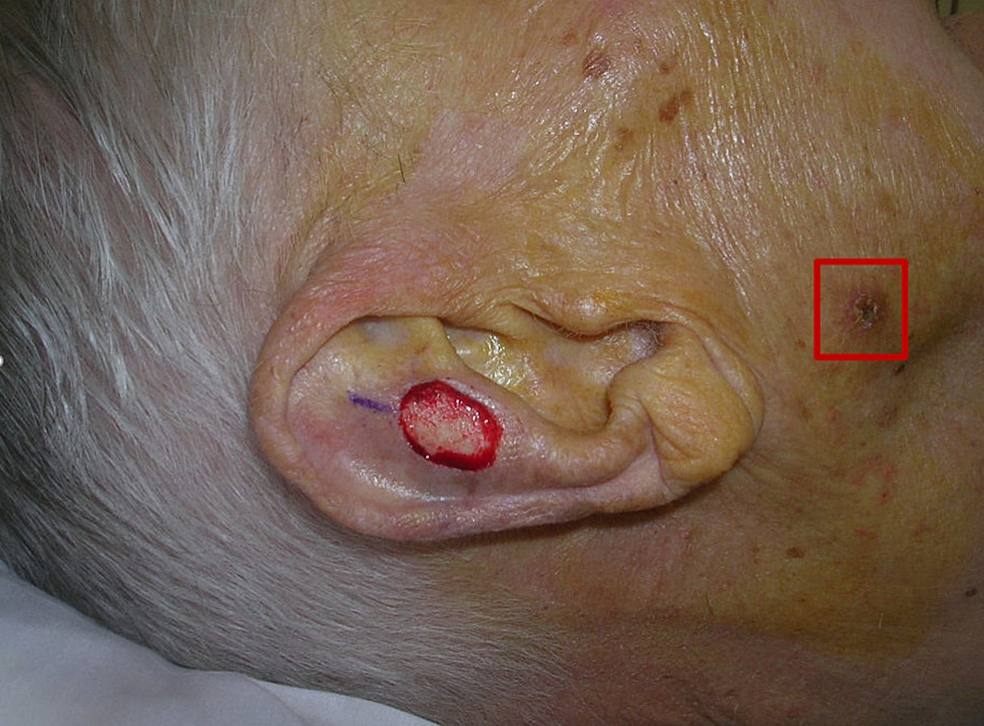
Unrecognized squamous cell carcinoma (at least in situ) on the right cheek at the time of Mohs surgery.

**Figure 3:**
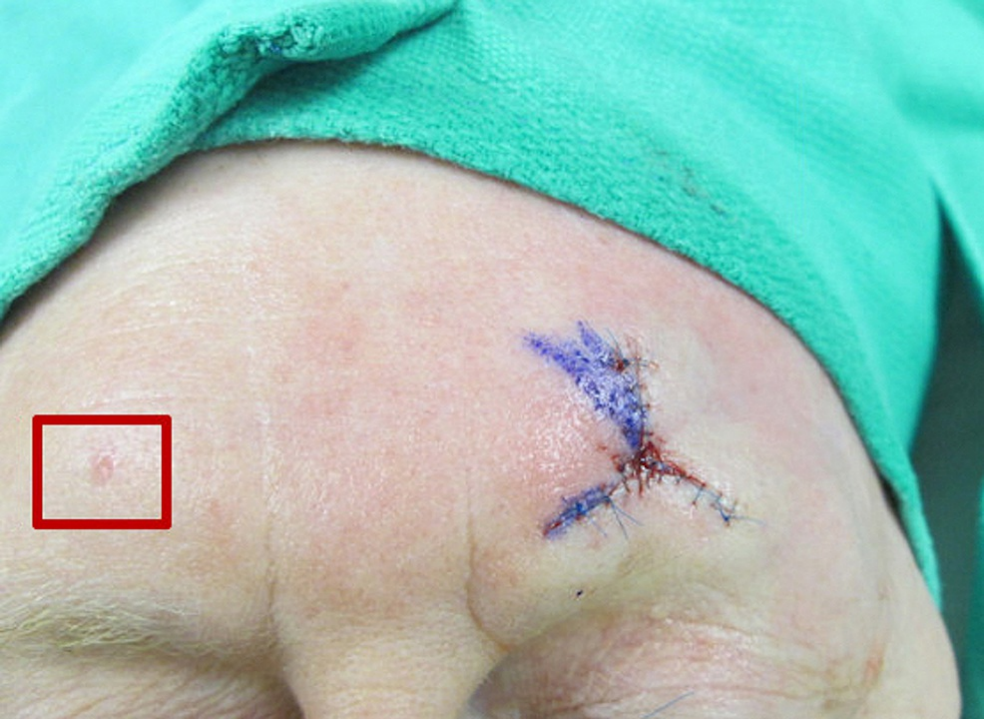
Unrecognized basal cell carcinoma on the right forehead at the time of Mohs surgery.

Our results suggest that dermatologic surgeons may overlook skin cancers that are clinically apparent. Figures 2, 3 demonstrate examples of tumors that were unrecognized by the dermatologic surgeon at the time of MMS. We were able to identify these unrecognized tumors in up to 4.6% of patient encounters on retrospective photograph review. We believe that cognitive bias, specifically tunnel vision, may be a factor contributing to this phenomenon.

Tunnel vision is a challenge to overcome that depends on minimizing the cognitive biases that produce it [10]. Metacognition (thinking about thinking) may allow surgeons to recognize the limits of their cognitive faculties in order to find a new perspective and engage in self-critique to prevent intuitive mistakes [16]. Metacognition forms the basis of cognitive forcing strategies that enable practitioners to make unbiased decisions [17]. However, we want to emphasize that an intense focus impervious to distraction is critical for a surgeon to perform an operation successfully and minimize error [18]. Therefore, the overall concept of tunneling is neither inherently good nor bad. Rather, surgeons must strike a balance between the level of attention required to perform surgery and potentially falling prey to tunnel vision bias. Bearing this in mind, we present several practical cognitive forcing strategies to better equip medical providers to combat tunnel vision bias.

The first step for dermatologic surgeons is to realize that they may have a blind spot. Only then can they implement the strategies to mitigate the effects of cognitive bias (Table 2) [15]. A surgeon’s current perspective assumes that a patient presenting for surgery has already been thoroughly examined by a dermatologist, and therefore no additional skin cancers are present. Yet, our study suggests that there is a 16% (178/1,110) chance of an MMS patient having a second, clinically apparent tumor at any given encounter. Dermatologic surgeons should alter their perspective to assume the baseline position that there any patient presenting for surgery could have another undiagnosed skin cancer (Table 2) [19].

**Table 2:** Cognitive Forcing strategies to combat tunnel vision bias (adapted from Lowenstein).

Cognitive Forcing Strategy	Practical Application
Change the base case assumption	Do not assume that a return or referred patient is fully worked up. Rather, assume undiagnosed secondary problems are present that require thoughtful evaluation.
Use a checklist	Assess regional skin in the field of surgery as a routine part of each patient visit to help minimize missing diagnoses.
Engage in self-assessment	Review prior objective evidence- photographs, biopsy reports, lab tests- for signs of medical error that may have been overlooked due to heuristics. Keep a log of these diagnostic errors to periodically review and prevent future cognitive bias.
Avoid fatigue and reduce cognitive strain	Consider small glucose snacks to refill one’s mental reserve, get adequate sleep, limit interruptions, allocate ample time for cognitively demanding cases.
Alter the practice environment	Build extra time into your schedule to allow for feedback, checklists, and reflective self-evaluation. Consider using scribes and information technology to allow more focused energy on patient care.

Surgeons should consider changing the external environment to facilitate finding tumors that are not yet diagnosed but present on exam. This could be as simple as performing a regional exam as part of a checklist when evaluating patients. Using a checklist ensures the exam is completed during each patient encounter [19]. Clinicians may implement an internal self-assessment when a patient returns for an additional procedure. Surgeons can do this by viewing the operative photographs in order to determine if the “new” lesion was actually visible at the previous encounter.

Cognitively demanding tasks, such as those required to overcome cognitive heuristics, deplete one’s mental reserve for exerting self-control in a subsequent task. In turn, the more mentally drained a person becomes, the more likely it is that the individual relies upon cognitive heuristics [1]. Baumeister showed that increasing the levels of glucose in the brain by consuming sugar decreases the number of intuitive errors that a fatigued person commits [20]. Therefore, a simple biohack (using science to optimize one’s biology), that a cognitively fatigued physician can employ is consuming a small glucose snack to refuel the brain in order to better combat cognitive bias such as tunnel vision bias (Table 2) [21].

As seen in Table 1, there was significantly more immunosuppressed patients in the recognized cancer event group compared to the unrecognized cancer event group (39% vs. 14%, p=0.001). Consequently, the significantly more SCCs in the recognized cancer event group compared to the unrecognized cancer event group (74 (62%) vs. 16 (32%), p<0.001) likely stems from having significantly more immunosuppressed patients in the recognized group (39% vs. 14%, p=0.001). One explanation for the increased recognition of tumors in immunosuppressed patients is that providers may understand that this group is at a higher risk of developing skin cancer. As a result, the clinicians may deliberately slow down (engaging System 2 thinking) when dealing with this high-risk population in order to identify additional skin cancers at the time of surgery. This may be an example of how System 2 is ultimately in charge and can override System 1 to help avoid committing a cognitive bias [1]. However, an alternative explanation is that immunosuppressed patients are more cognizant of tumors (large, rapidly growing tumors or having a history of numerous skin cancers) and would therefore be more likely to bring concerning lesions to the attention of the provider, which would prompt biopsy. 

The dermatologic surgeons in our study recognized over twice as many clinically apparent tumors compared to those that they overlooked (127 recognized versus 51 unrecognized), which may suggest they are engaging System 2 style thinking. Clinicians should strive to extend this higher order, reflective thinking to all patients that they see in the clinic. Lastly, there were no significant differences between the unrecognized and recognized cancer event groups in regard to high-risk histopathology or Mohs interval until definitive treatment for unrecognized tumors (Table 1). 

Limitations

The study limitations include the retrospective nature and single tertiary care center, which limits generalizability. This study may underestimate the true number of unrecognized tumors due to a lack of photographs for all relevant locations. Additionally, the quality of photographs limits our ability to identify all suspicious lesions. The current methodology could not include patients who were seen by their local providers to manage subsequent skin cancers and did not return to the MMS clinic. We cannot be certain that all tumors marked as “unrecognized” were truly overlooked. Although our clinical practice is to biopsy suspicious lesions, there may have been some patient scenarios where an informal discussion was had with the patient recommending that the patient seeks care at the next appointment with their referring general dermatologist or where biopsies were deferred per patient preference. While we believe that cognitive bias may contribute to the study’s findings, the retrospective nature makes this assertion less robust.

Our study group of patients all had at least two Mohs surgeries, which suggests a higher tumor burden compared to the average patient. This is supported by the relatively high number of immunosuppressed patients in the study (14% and 39% for the unrecognized cancer event and recognized cancer event groups, respectively). Therefore, these patients may be more likely to have secondary tumors at the time of presentation for Mohs surgery. This may affect the generalizability of our study. Our recommendations for combatting deleterious cognitive heuristics such as tunnel vision bias are not based on randomized trials but rather on psychological principles and practical considerations. We recommend additional prospective or multi-center studies in the future to further elucidate the role of tunnel vision bias in overlooking clinically apparent tumors during dermatologic surgery.

## Conclusions

Our study suggests that Mohs surgeons may be overlooking adjacent skin cancers when focusing only on the tumor being surgically treated during MMS. Tunnel vision bias may account for part of this phenomenon. While a high degree of focus is necessary for Mohs surgery, the indiscriminate focus may contribute to overlooking a tumor. Delays in care from not recognizing tumors could result in increased morbidity, especially for high-risk tumors and high-risk groups. Dermatologic surgeons should be aware of the likelihood of a second clinically apparent skin cancer being present at any given patient encounter. Surgeons are not uniquely susceptible to medical errors due to tunnel vision bias. Rather, tunnel vision bias may impact all areas of dermatology including surgery, dermatopathology, medical dermatology, laser therapy, cosmetic procedures, and specialty clinics where clinicians specifically hone in on one disease process. We advocate for all providers to be aware of the potential presence of regional disease in a treatment area in order to avoid potential patient morbidity associated with overlooking tumors.

An important secondary aim of our study is to remove the stigma of tunnel bias-related errors. These errors stem from mental processes hard-wired within the human brain, and dermatologists should seek strategies to mitigate cognitive biases in order to improve patient care.
